# Therapeutic hypothermia after perinatal asphyxia in Vietnam: medium-term outcomes at 18 months – a prospective cohort study

**DOI:** 10.1136/bmjpo-2023-002208

**Published:** 2024-02-22

**Authors:** Hang Thi Thanh Tran, Ha Thi Le, Dien Minh Tran, Giang Thi Huong Nguyen, Lena Hellström-Westas, Tobias Alfven, Linus Olson

**Affiliations:** 1 Department of Global Public Health, Karolinska Institute, Stockholm, Sweden; 2 Neonatal Care Center, Vietnam National Children's Hospital, Ha Noi, Viet Nam; 3 Vietnam National Children's Hospital, Ha Noi, Viet Nam; 4 Department of Rehabillitation, Vietnam National Children's Hospital, Hanoi, Vietnam; 5 Department of Womens and Childrens Health, Uppsala University, Uppsala, Sweden; 6 Sachs’ Children and Youth Hospital, Stockholm, Sweden; 7 Department of Women's and Children's Health, Karolinska Institutet, Stockholm, Sweden

**Keywords:** neurology, rehabilitation, neonatology

## Abstract

**Aim:**

To determine neurodevelopmental outcome at 18 months after therapeutic hypothermia for hypoxic-ischaemic encephalopathy (HIE) infants in Vietnam, a low-middle-income country.

**Method:**

Prospective cohort study investigating outcomes at 18 months in severely asphyxiated outborn infants who underwent therapeutic hypothermia for HIE in Hanoi, Vietnam, during the time period 2016–2019. Survivors were examined at discharge and at 6 and 18 months by a neonatologist, a neurologist and a rehabilitation physician, who were blinded to the infants’ clinical severity during hospitalisation using two assessment tools: the Ages and Stages Questionnaire (ASQ) and the Hammersmith Infant Neurological Examination (HINE), to detect impairments and promote early interventions for those who require it.

**Results:**

In total, 130 neonates, 85 (65%) with moderate and 45 (35%) with severe HIE, underwent therapeutic hypothermia treatment using phase change material. Forty-three infants (33%) died during hospitalisation and in infancy. Among the 87 survivors, 69 (79%) completed follow-up until 18 months. Nineteen children developed cerebral palsy (8 diplegia, 3 hemiplegia, 8 dyskinetic), and 11 had delayed neurodevelopment. At each time point, infants with a normal or delayed neurodevelopment had significantly higher ASQ and HINE scores (p<0.05) than those with cerebral palsy.

**Conclusion:**

The rates of mortality and adverse neurodevelopment rate were high and comparable to recently published data from other low-middle-income settings. The ASQ and HINE were useful tools for screening and evaluation of neurodevelopment and neurological function.

**WHAT IS ALREADY KNOWN ON THIS TOPIC**

**WHAT THIS STUDY ADDS**

- Rates of mortality and delayed neurodevelopment were high in this group of infants with HIE who were born at term and treated with hypothermia in a low-middle-income setting.
- The Ages and Stages Questionnaire and the Hammersmith Infant Neurological Examination are simple and easy tools in the early identification of those infants needing a specific rehabilitation programme.

**HOW THIS STUDY MIGHT AFFECT RESEARCH, PRACTICE OR POLICY**

- The neurodevelopmental results in this study confirm that hypothermia-treated infants require close follow-up in a standardised multidisciplinary programme. There should be more studies to investigate the role of therapeutic hypothermia in low-middle-income country settings as well as longer-term outcomes of affected infants.

## Background

 Hypoxic-ischaemic encephalopathy (HIE) resulting from a lack of oxygen and blood flow to the brain after delivery is one of the leading causes of neonatal deaths. It is estimated to affect 1–2 per 1000 newborns in high-income countries (HICs), and 10–20 in low-middle-income countries (LMICs).^1^ Newborns with mild encephalopathy appear to have normal neurocognitive outcomes,^2 3^ while those surviving with severe encephalopathy are more likely to have profound disability.^4^

Therapeutic hypothermia (TH) has been proven in randomised controlled trials (RCTs) to reduce mortality and neurological deficits at 18 months in babies with moderate to severe HIE.^5^ Several cohort studies of asphyxiated children show that the disability rate among surviving newborns with moderate to severe HIE can be as high as 40%–100% if they are not cooled.^2 6^ To improve health outcomes for children and their families, early diagnosis and treatment of developmental delays and disorders are crucial.^7^

Outcomes for newborns treated with TH in LMICs have been documented in a small number of trials.^8^ Studies from LMIC with positive results handled these newborns in a neonatal intensive care unit (ICU) setting comparable to that of HICs^9^ but only focused on neurodevelopmental outcomes for a limited time frame of 6–12 months. Only four RCTs from China and India reported neurodevelopmental outcomes beyond 12 months of age, and all four indicated the favourable impact of TH.^10^,^13^ A recently published multicentre RCT of TH for moderate or severe neonatal encephalopathy, the HELIX trial, comprising a total of 7 study sites and 408 infants in LMICs showed that TH did not reduce the combined outcome of death or disability at 18 months; hence, it was suggested that TH should not be recommended as a treatment for HIE in such settings.^14^

The aim of the present observational study was to determine outcomes, that is, mortality and neurodevelopmental outcomes at 18 months in survivors, in transported asphyxiated neonates treated with TH in Hanoi, Vietnam.

## Methods

### Study design and participants

This study was a prospective cohort study with assessment of neurodevelopmental outcomes at 6 and 18 months in survivors with HIE who underwent TH at Vietnam National Children’s Hospital (VNCH). The infants were born in 2016–2019 and delivered in district or provincial hospitals and required resuscitation for at least 10 min after delivery. They were assessed as having moderate to severe HIE at the hospital where they were delivered and were randomised into two groups: transport with passive cooling (undressed, no heating) or on a mattress made of phase-change material (PCM) (Medical Cooling Sweden AB; by TST AB, Kinna, Sweden). The inclusion criteria for TH were based on modified TOBY trial criteria,^15^ including a birth history indicating perinatal asphyxia and need for resuscitation during the first 10 min. However, blood gases could not be measured at delivery in any of the referring hospitals, and Apgar scores were frequently missing. The decision to transport the infants for TH was made by the attending neonatologist at VNCH after telephone consultation from the referral hospital. Exclusion criteria for TH included >6 hours of age at the time of admission at VNCH, being <36 weeks, and/or having significant congenital anomalies.

### Patient and public involvement

Neither patients nor the public were involved in the design of the study, nor conducting, reporting or disseminating plans of our research.

### Treatment

All newborns were subjected to TH using a PCM mattress with a target rectal temperature of 33.5°C–34.5°C for a period of 72 hours, followed by rewarming to normothermia at a rate of no more than 0.5°C per hour. On arrival (day 1), encephalopathy was classified as mild, moderate or severe using the modified Sarnat classification. Assessment of Thompson score was done daily during TH and at discharge. On days 1 and 7, a cranial ultrasound was conducted. Depending on the patient’s clinical condition, a brain MRI was performed after 7–10 days. All of the patients received standard critical care in addition to the cooling procedure.

### Follow-up and neurodevelopmental assessment

At discharge, a comprehensive Hammersmith Neonatal Neurological Examination was performed by a neonatologist, in addition to normal clinical discharge procedures. Follow-ups at the clinic were scheduled at 6 months and 18 months of age. During these follow-ups, neurodevelopment and neurological assessments were performed by a team consisting of a neonatologist, a neurologist and a rehabilitation doctor, all of whom were blinded to the infant’s clinical severity during hospitalisation.

**Figure 1:**
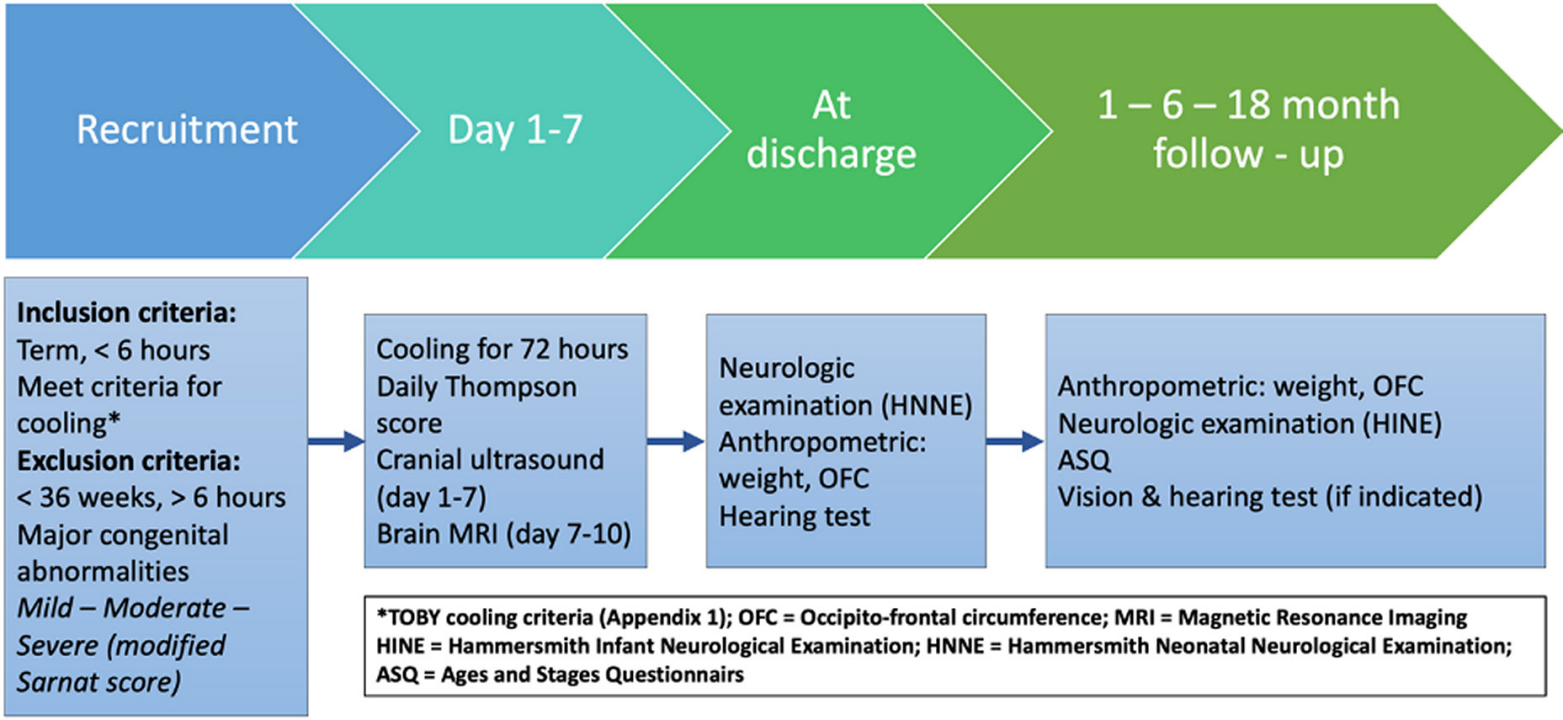
Study flow.

At each follow-up a Ages and Stages Questionnaire (ASQ) evaluation was also performed. A rehabilitation doctor asked the parents questions and examined the child’s function in the following areas: communication, gross motor, fine motor, social and problem-solving skills. After completing the ASQ and comparing the results with prespecified cut-off scores, those who were in need were indicated for physical therapy. If a child had ≥2 subscores below minus 2 SD below the mean in any of the ASQ domains, he/she was determined as high risk for developmental delay.^16^ The Hammersmith Infant Neurological Examination (HINE) was used for clinical neurological examination and consisted of five parts: cranial nerve function, posture, movements, tone and reflexes, with a total of 26 items; each item could be scored from 0 to 3, resulting in a maximum total score of 78.^17^,^19^ The diagnosis of cerebral palsy (CP) was made at 18 months according to Bax *et al* diagnostic criteria^20^ during a clinical examination. Screening visual and hearing tests were indicated during follow-up by a specialist doctor if needed. Figure 1 shows the study’s participants and the study flow.

### Outcomes

The primary outcome was developmental outcome at 18 months of age: normal development, developmental delay or CP. A child was identified as having ‘normal development’ if, at the time of assessment, he/she progressed through predictable developmental phases; and those with delay in meeting developmental milestones in one or more streams of development were defined as ‘developmental delay’. Severe disability or CP was defined as a permanent, non-progressive disorder in the development of movement and posture and the diagnosed was based on a combination of clinical history, neuroimaging evidence and neurological exams. Secondary outcomes included the ASQ score, HINE score, and vision, hearing, and other impairments.

### Statistical analysis

Birth weight and gestational age are reported as mean and SD. Differences among groups were analysed with a one-way analysis of variance test. ASQ and HINE scores are reported as median and IQR (for data that were not normally distributed) for the four examination ages. The ability of the ASQ and HINE to identify developmentally delayed children was then determined using the following test characteristics: sensitivity, specificity, positive predictive value (PPV), negative predictive value (NPV), over-referral (false positives) and under-referral (false negatives) using the MedCalc Software, diagnostic test evaluation calculator (https://www.medcalc.org/calc/diagnostic_test.php). The level of significance was set at p<0.05. We used SPSS statistical software (Version 20) for the analysis.

## Results

**Figure 2:**
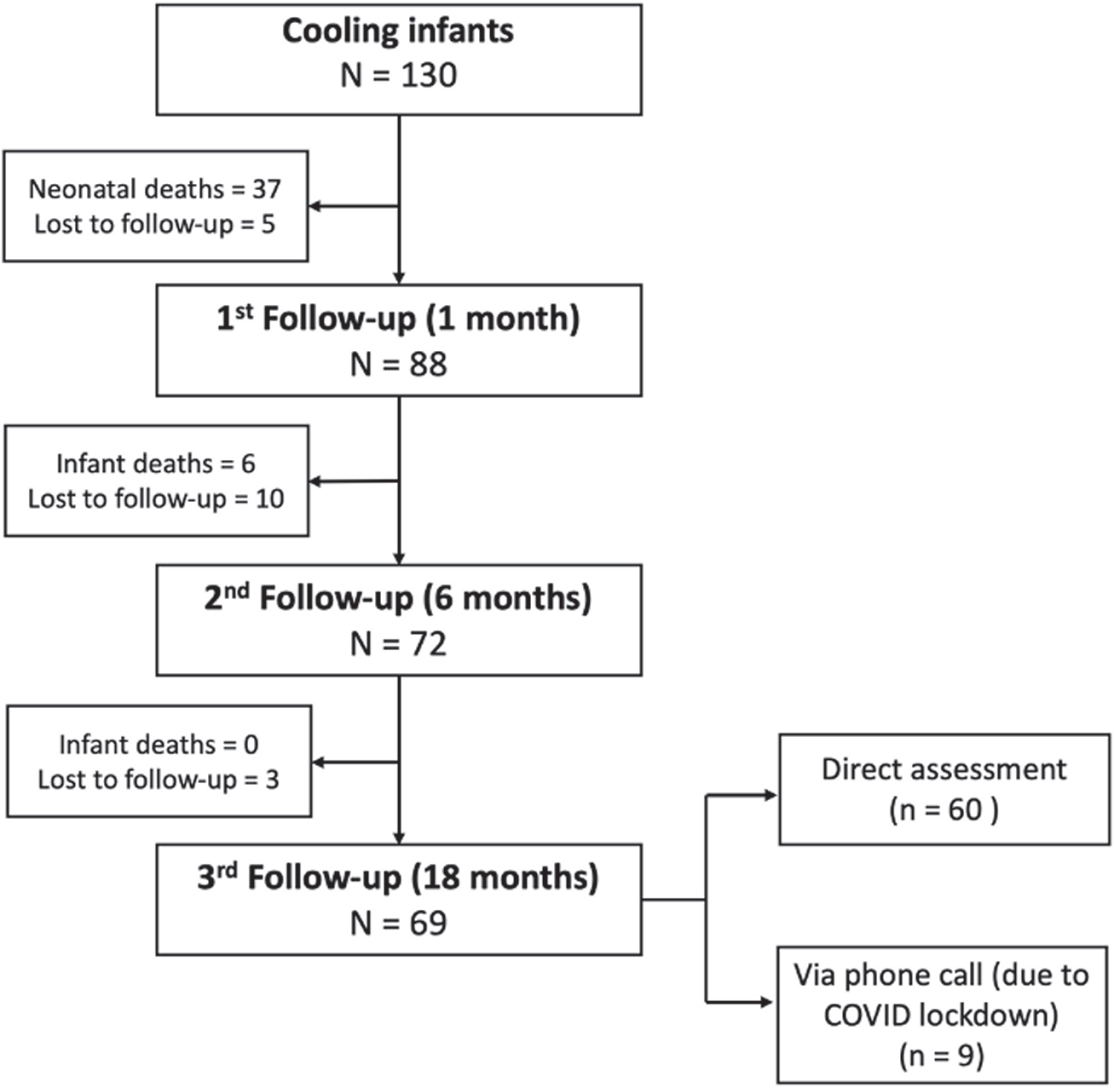
Recruitments, follow-up.

Between June 2016 and December 2019, a total of 130 infants underwent TH treatment at VNCH. In total, 43 (33%) infants died during hospitalisation and later in their infancy: 38 due to severe encephalopathy, 3 due to persistent pulmonary hypertension and 2 due to pneumothorax. During the 18-month period follow-up, 18 patients were lost to follow-up: 8 were uncontactable, 5 refused to come due to the long travel time and 5 due to the severity of their infants’ condition. The final evaluation at 18 months was performed for 69 patients (79% of the survivors), in which only 60 could come to the hospital, and 9 were interviewed via a phone call, due to the COVID-19 lockdown in Vietnam. The number of infants included at each time point is summarised in figure 2.

### Characteristics of population

The background characteristics of the population, as related to outcomes—dead, normal neurodevelopment, developmental delay or CP among survivors at 18 months—are presented in table 1. Of the 19 infants with CP, 8 had diplegia, 3 showed hemiplegia and 8 dyskinetic type. There were no differences in terms of gestational age or birth weight between the three outcome groups. During hospitalisation, shock requiring inotrope use was recorded for 36 patients (28%) and end-organ failure, affecting kidney and liver, were diagnosed in 66 patients (51%). At 18 months, none of the children was diagnosed with hearing and/or visual impairments.

**Table 1:** Background characteristics of the study cohort

	Dead(n=43)	Survivors at 18 months (n=69)			P value
		Normal development (n=39)	Developmental delay (n=11)	Cerebral palsy (n=19)	
Male sex, n (%)	26 (60)	32 (78)	7 (64)	15 (79)	0.253
GA, mean(min-max), weeks	38.8 (35–42)	38.9 (36–41)	39.5 (38–40)	39.0 (36–40)	0.418
BW, mean(min-max), gram	2968 (2100–3900)	3196 (2700–4000)	3245 (2600–3900)	3163 (2100–4000)	0.007*
Sarnat stage III, n (%)	35 (81)	26 (68)	7 (70)	16 (76)	0.157
Temp on admission, °C	34.7 (0.9)	35.1 (0.9)	35.0 (0.9)	34.6 (0.7)	0.054
Time start TH, h	4.2 (1.2)	3.8 (1.2)	4 (1.2)	4.9 (1.3)	0.001*
aEEG, flat or burst suppression first 12 hours, n†	31/35 (89)	22/35 (63)	9/11 (88)	17/19 (89)	0.015*
Seizures, n (%)	12 (28)	5 (13)	3 (30)	5 (24)	0.062
Neonatal complications					
Sepsis, n (%)	4 (9.3)	0	2 (20)	3 (14)	0.673
Shock, n (%)	15 (35)	12 (31)	3 (27)	6 (31)	0.045*
End-organ failure, n (%)	27 (63)	15 (39)	8 (73)	16 (84)	0.647
Ventilation, n (%)	43 (100)	35 (90)	11 (100)	19 (100)	0.654
PPHN, n (%)	3 (7)	0	1 (10)	2 (9)	0.076
MRI abnormal at 7–10 days‡	10/35	10/36	6/11	19/19	0.000*
Basal ganglia/PLIC, n (%)	3 (9)	3 (8)	2 (20)	7 (37)	
Diffuse WMI, n (%)	4 (11)	5 (14)	3 (27)	10 (53)	
Other (atrophy, IVH), n (%)	3 (9)	2 (5)	1 (9)	2 (10)	

Values are numbers, percentages (%), ranges and mean (SD). Groups differences were analysed with a one-way ANOVA test, and a p<0.05 was considered to be statistically significant.

* sStatistically significant.

† aEEG data isare not available for all patients, total 100/130 patients got aEEG.

‡ Denominator was the number of infants who got MRI.

aEEGAmplitude integrated electroencephalographyANOVAanalysis of varianceBWbirth weightGAGestation agePLICposterior limb of the internal capsulePPHNPersistent pulmonary hypertension of newbornTHtherapeutic hypothermiWMIWhite Matter Injury

Abnormal MRI findings (basal ganglia injury or abnormal signal in the posterior limb of the internal capsule (PLIC), diffuse white matter injury (WMI), intraventricular haemorrhage grades III–IV, brain atrophy) were present in 45 out of 101 patients who underwent MRI and were significantly associated with adverse outcome. All children who developed CP had abnormal MRIs with basal ganglia/PLIC abnormalities in 37% and WMI in 53%.

### Infant neurodevelopment screening

Table 2 provides details of the ASQ scores at each time point. A progressive increase in ASQ scores was observed until 18 months. At each time point, infants with a normal or delayed neurodevelopment had significantly higher ASQ scores (p<0.05) than those with CP, but there were no differences between infants with normal and delayed neurodevelopment.

**Table 2:** Results of the Ages and Stages Questionnaire at 6 and 18 months divided in subsection scores, showing mean (SD) for the different outcomes

Age	Communication	Gross motor	Fine motor	Problem-solving	Personal social
6 months					
Normal	45 (11.0)	41.4 (11.5)	42.3 (8.3)	44.5 (13.5)	44.4 (12.3)
Developmental Delay	50.0 (11.0)	45.5 (8.8)	45.0 (7.4)	50.0 (11.0)	45.9 (10.9)
CP	21.6 (9.1)	15.8 (12.7)	16.8 (13.8)	20.8 (13.3)	25.5 (10.0)
18 months					
Normal	43.2 (13.4)	38.3 (15.2)	39.9 (13.1)	42.1 (16.1)	40.7 (15.4)
Developmental Delay	40.0 (18.0)	35.9 (18.1)	35.5 (14.6)	37.7 (19.7)	34.5 (14.7)
CP	16.8 (13.3)	9.5 (16.3)	13.4 (14.9)	9.2 (11.9)	15.3 (12.5)

Values are mean (SD) scores.

CPcerebral palsy

### Infant neurological examination

**Figure 3:**
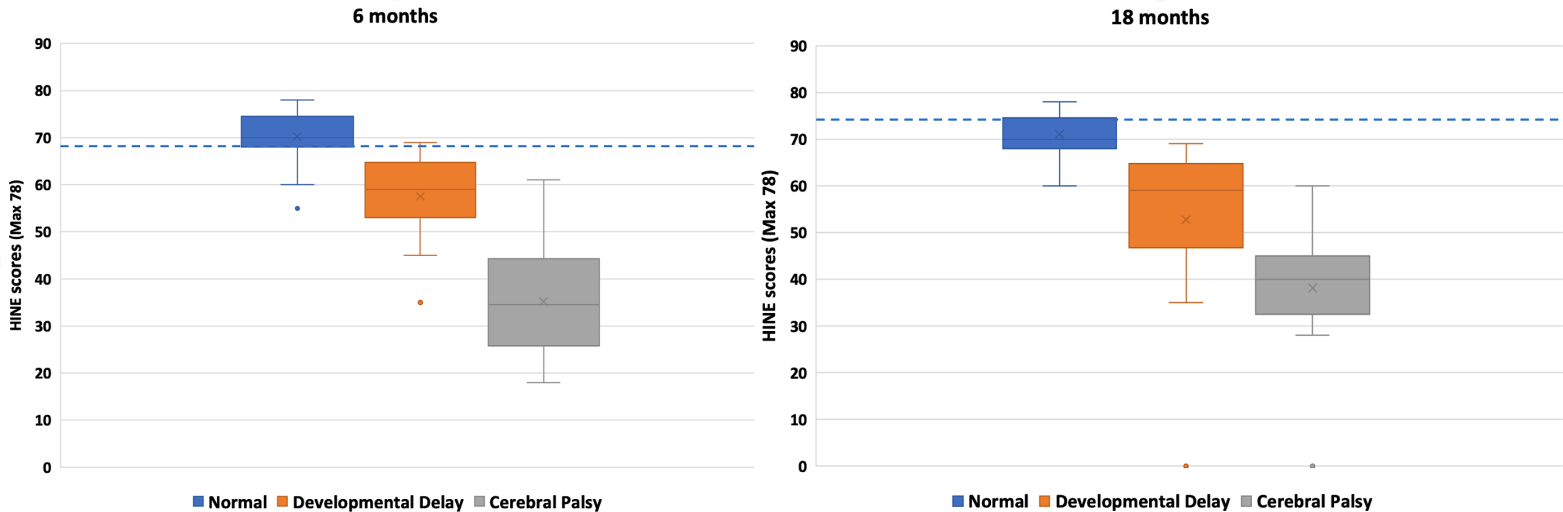
HINE scores at 6-month and 18-month follow-up. HINE, Hammersmith Infant Neurological Examination.

At 6 months of age, all infants with normal outcome, at the age of 18 months, scored above 50 on the HINE (figure 3). The mean (SD) total scores for the HINE results at 6 months were 70 (6) for the normally developing children, 57 (10) in the delayed and 35 (11) in the children with CP (p<0.001). These results can be compared with the global reference score for normal development, 68 (range 54–76). The corresponding scores at 18 months were 71 (7), 57 (4) and 39 (8) (p<0.001) for group differences, and compared with the global reference score of 74 (65–78)^21^ (figure 3).

**Figure 4:**
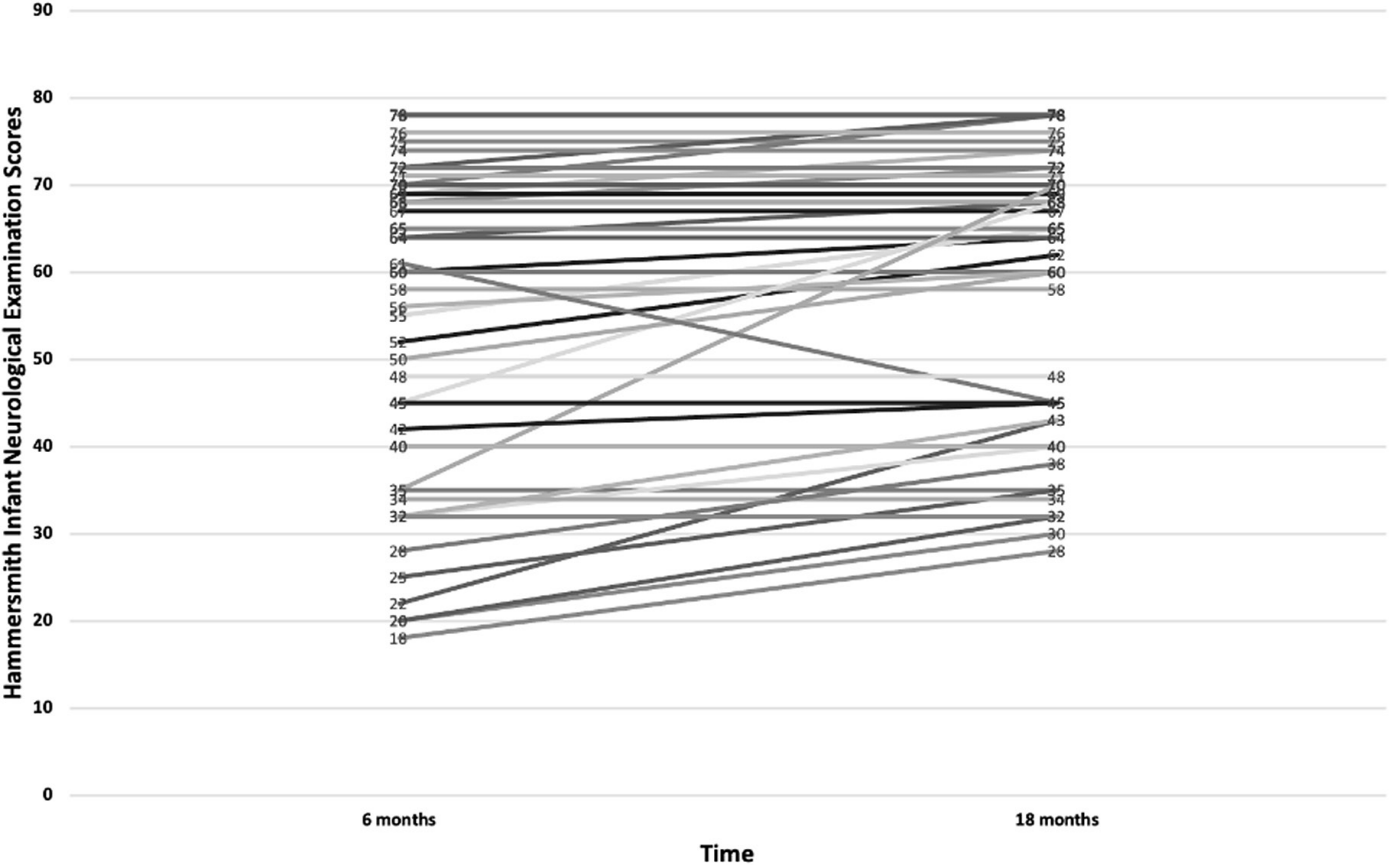
Individual HINE scores at 6 and 18 months. HINE, Hammersmith Infant Neurological Examination.

At 6 months, a HINE score below 40 predicted CP at 18 months with sensitivity of 68%, specificity of 98%, PPV 93% and NPV 89%, and with an accuracy of 90% (95% CI 81% to 96%). On the other hand, a score above 68 predicted normal development at 18 months with a sensitivity of 70%, specificity of 97%, PPV of 96% and NPV of 75%, and with an accuracy of 83% (95% CI 72% to 91%). In figure 4, the individual infants’ HINE scores can be followed from 6 to 18 months. The cut-off points for predicting good and poor outcomes were adapted from Romeo *et al* study.^22^

## Discussion

This study confirms the significant risk of mortality or long-lasting effects on neurodevelopment in infants with HIE born at term and treated with hypothermia in a low-middle-income setting.

In a recent meta-analysis of 29 RCTs of TH for HIE, including multiple income settings, Mathew *et al* showed that the combined proportion of death or neurological disability at 18–24 months in TH and normothermia groups was 45% and 57%, respectively.^23^ The results from our study, with combined infant mortality and severe neurological disability of 55% in infants treated with TH, were comparable to both the results in the randomised HELIX trial, in part performed during the same time as this observational study, and to a case–control study in Nepal, performed almost 20 years earlier in the mid 1990s.^14 24^ The outcomes might be affected by inadequate prenatal care, suboptimal initial resuscitation after delivery, which would prolong hypoxia and lengthy transit durations, which were on average 3–4 hours. Similar conditions were also reported in other studies in LMICs with poorer outcomes than in HICs.^14^

In this study, more than half of all infants had severe adverse short-term events. Compared with another study in a similar setting, the proportion of infants with haemodynamic shock and end-organ failure were comparable, although this study had slightly lower rates of PPHN and sepsis.^25^ Our ICU care offered initial mechanical ventilation for those who required ventilatory support, intravenous nutrition, standard monitoring including ECG, heart rate, oxygen saturation, and aEEG, and treatment of clinical and electrographical seizures with phenobarbital.

In infants who suffer from moderate to severe HIE, early neonatal predictive indicators of neurological outcomes together with early detection of delayed or abnormal development combined with timely intervention, are essential for rational clinical decisions in order to optimise the neurodevelopment of affected infants. Several neurological assessment tools have been used over the years to forecast outcomes of high-risk infants. The largest study to date, which evaluated 903 high-risk infants at 3 months, using the HINE, reported a sensitivity of 98% and a specificity of 94% for the development of CP.^22^ In this study, a HINE score below 40 at 6 months predicted CP at 18 months with a lower sensitivity, 68%, but a similar high specificity, 98%. A systematic review has shown that diagnosis of CP can be accurately made before 5 months with magnetic resonance imaging (86%–89% sensitivity).^26^ In this study, we could also see differences in MRI results between the different neurological outcome groups. Furthermore, abnormal HINE scores in combination with abnormal MRI are even more accurate than single clinical assessments in isolation.^17 27^ Another study of 94 infants with mild to severe HIE in Vietnam also showed that grey matter injuries, white matter injuries and cerebellar lesions were associated with higher mortality and CP in survivors.^28^

Difficulties in following children over time in repeated long-term follow-up of high-risk infants are prevalent in all settings, in LMICs as well as in high-income settings.^29^ Consequently, a parent-based evaluation that can be conducted at home is easier, comes at a lower cost and is less time-consuming for both the parent and the healthcare system. The advantages of ASQ, in screening for high-risk infants in comparison to other available scales, are its cost-effectiveness, easy implementation and high validity. Consequently, ASQ has been translated into numerous languages and is used all over the world.^30^,^32^ Lindsay *et al* showed that with an ASQ completed by parents, it was possible to identify children with severe developmental delay at 12–14 months, with excellent sensitivity at 92% and a specificity of 95%, PPV of 92% and NPV of 95%.^29^ Also, there are clear differences in this study between children with normal development and those with neurodevelopment disabilities, where the usage of ASQ has been helpful as a tool to identify these differences.

This study was limited by the 18-month follow-up period, which was chosen because it was more feasible for parental compliance. It is possible that some infants with neurodevelopment delay developed a normal neurological outcome after this age, or that some of them might be diagnosed with mild CP. The diagnosis and grading of neurodevelopment outcome in the current study was clinical since no other scale or tool was available in this setting.

Consequently, assessment at subsequent ages might provide a more precise assessment of the final neurological and neurodevelopment outcome. Longer-term follow-up in LMIC cohorts would help to predict lifelong outcome since children who were cooled were shown to be less prepared for school than their typically developing peers in a UK cohort that followed them until preschool age.^33^ Further, 20% of the survivors were lost to follow-up, mostly due to being uncontactable or unwilling to travel the long distance to the research site. It is possible that those who defaulted could have had a higher prevalence of developmental delays and/or CP, and this is a potential source of bias in the results.

### Conclusion

The rates of mortality and adverse neurodevelopment in this low-middle-income setting were high, comparable to results from other similar settings. Although not formally evaluated, we found that the ASQ and HINE were useful tools for early screening of neurodevelopment and assessment of neurological function in these children.

## Data Availability

Data are available on reasonable request.
