# Supplementary material for: Therapeutic hypothermia after perinatal asphyxia in Vietnam: medium-term outcomes at 18 months – a prospective cohort study
Source: BMJ Paediatr Open. 2024 Feb 22;8(1):e002208. doi: 10.1136/bmjpo-2023-002208 (PMC10882320; doi:10.1136/bmjpo-2023-002208)
Supplement: Supplementary file 1 [file bmjpo-2023-002208.reviewer_comments.pdf]

PEER REVIEW HISTORY

BMJ Paediatrics Open publishes all reviews undertaken for accepted manuscripts. Reviewers are asked to complete a checklist review form and are provided with free text boxes to elaborate on their assessment. These free text comments are reproduced below.

ARTICLE DETAILS

**TITLE (PROVISIONAL)**	Therapeutic hypothermia after perinatal asphyxia in Vietnam - medium-term outcomes at 18 months: a prospective cohort study
**AUTHORS**	TRAN , HANG THI THANH<br>Le, Ha Thi<br>Tran, Dien Minh<br>Tran, Hanh Thi Phuong<br>Hellström-Westas, Lena<br>Alfven, Tobias<br>olson, linus

VERSION 1 – REVIEW

**REVIEWER**	Medha Goyal
**REVIEW RETURNED**	03-Aug-2023

**GENERAL COMMENTS**	An important topic explored by the team!<br>However following changes are needed to improve the quality of paper before it can be considered for publication.<br><br>1. Manuscript needs grammatical corrections and uniform usage of terminology such as infants/babies/newborn/neonate etc.<br>2. Need to use neurodevelopment and neuromotor outcomes as relevant based on the method used for assessment<br>3. Table 1:<br>- RR/OR/Confidence interval for p values<br>- Key details of TH: Hours of initiation, any temperature deviations, temperature at admission, need of ventilation, circulatory dysfunction, coagulopathy, renal failure, end organ dysfunction needs to be compared as they impact the neurodevelopment outcomes<br>- Apgars, cord gases for baseline comparison<br>- Growth restriction/SGA<br>4. How has the sensitivity, specificity, PPV and NPV established. Describe the gold standard used for comparison.<br>5. To describe other studies that have been carried out in LMICs and have looked at short-term outcomes of TH for comparison (Mascarenhas D, Goyal M, Nanavati R, Kirthana SB, Subhadarsini S. Short-term outcome and complications of therapeutic hypothermia in neonates with moderate-to-severe hypoxic ischaemic encephalopathy: a single-centre retrospective observational study in a hospital in Mumbai, India. Paediatr Int Child Health. 2023 Feb 8:1-10. doi:

	10.1080/20469047.2023.2171762. Epub ahead of print. PMID: 36755411.)<br>6. Hearing and vision assessments results not described in the results section<br>7. When was cerebral palsy diagnosed? Not clear in methodology<br>8. The neurological impairment described are substantially high hence needs more description of therapeutic hypothermia protocol in the unit<br>9. Conclusion needs to be modified in abstract as current study has not evaluated risk factors for NDI.<br>10. Data on CFM recordings

**REVIEWER**	Dr. Peter Flom<br>Peter Flom Consulting
**REVIEW RETURNED**	12-Aug-2023

**GENERAL COMMENTS**	I confine my remarks to statistical and methodological aspects of this paper.<br><br>I wonder why more sophisticated analyses weren't done on such useful data. Having scores at multiple time points would let you trace the path of each baby and you could use multilevel models to look at whether any covariates affect that path, or the sensitivity, specificity etc. If cooling worked better for boys, or something like that, that would be very good to know.<br><br>But I won't insist on it.<br><br>The analysis in Table 1 needs to be described in the text and mentioned in a footnote to the table.<br><br>In Table 2 at 6 months, how can mild delay be better than normal? Is this a typo? If not, it needs discussion. It also does not match figure 3.<br><br>It would also be nice to have figures showing infants over time. You could show time on the X axis and score on the Y axis and a line for each child. With your N you might want to divide this into two or more figures, either randomly or by some sensible criteria.<br><br>Peter Flom

**REVIEWER**	Dr. Shivshankar Diggikar<br>Ovum Women and Child Hospital, Neonatology
**REVIEW RETURNED**	31-Aug-2023

**GENERAL COMMENTS**	Comments to authors: Good job to collate the data which is difficult in LMIC setting.please see below l changes required.

	Title: Please mention what outcomes – short or long term, Neurodevelopmental to be specific in the title.<br><br>Abstract:<br>Line 51-52: Conclusion: Mortality and risk for adverse neurodevelopment were relatively high but comparable to other cohorts. Comparable to what cohorts ??<br>Line 53-56 The ASQ and HINE are helpful tools in the process of early diagnosis of infants at risk for cerebral palsy or delayed neurodevelopment.<br>Kindly stick to the results. The study is not about ASQ/HINE validation so remove this line and better to stick to the outcomes please. If you want to mention them in your objectives which doesn't state the same.<br>Introduction : Needs updating, use HIE or<br>Methods : Need more details<br>- Type of PCM used (Company and manufacturer name)<br>- Inclusion criteria as per TOBY used blood gas. Does the study centre used Blood gas for all infants ??<br>Results<br>- Baseline characteristics of all infants who underwent cooling needs to be presented total of 130 infants. The baseline data of survivors can be different table . or babies who died versus who survived which gives some info on the phenotype of HIE presentation and severity.<br>Table 1 : Please highlight the significant P values example : Neonatal seizures $p<0.05$ etc as legend<br>Is the P value given between two groups or three groups – Was ANOVA used ? if so mention in the legend<br>Use categorization of MRI abnormalities which are standard for HIE staging by Seeta shankaran et al Shankaran S, McDonald SA, Laptook AR, et al. Neonatal magnetic resonance imaging pattern of brain injury as a bio- marker of childhood outcomes following a trial of hypother- mia for neonatal hypoxic-ischemic encephalopathy. J Pediatr. 2015;167(5):987-993.e3. OR Diggikar S, Venkatagiri P, Kulkarni S, Sharma D, Chandra P. Clinical and Neuroimaging Profile of Neonates With Moderate-to-Severe Encephalopathy Treated With Therapeutic Hypothermia. Journal of Neonatology. 2022;36(4):268-274. doi:10.1177/09732179221130671<br><br>Discussion : Overall discussion is vague, need to be more specific. Needs modification in language and grammar.<br><br>Conclusion : Doesn't match the objectives stated. Needs to be changes

VERSION 1 – AUTHOR RESPONSE

Reviewer: 1

1. Manuscript needs grammatical corrections and uniform usage of terminology such as infants/babies/newborn/neonate etc.

Reply to reviewer: Thank you for this comment. We have checked the manuscript again and now refer to study participants as “newborn” immediately after birth, and then as “infants”.
The manuscript was already checked by a professional English editor; however, it has been edited by an English editor again.
2. Need to use neurodevelopment and neuromotor outcomes as relevant based on the method used for assessment

Reply to reviewer: Thank you for this comment. We have changed the text to distinguish ASQ as a measure of neurodevelopment and HINE and neurological examination for assessment of neuromotor outcome. In the result section, we have also put Infant Neurodevelopment and Neurological Examination results into two separated parts.

3. Table 1:
- RR/OR/Confidence interval for p values
- Key details of TH: Hours of initiation, any temperature deviations, temperature at admission, need of ventilation, circulatory dysfunction, coagulopathy, renal failure, end organ dysfunction needs to be compared as they impact the neurodevelopment outcomes
- Apgar’s, cord gases for baseline comparison
- Growth restriction/SGA

Reply to reviewer: Table 1 has been revised to include key details of TH as suggested, as well as p-values for all variables. We have conferred with our study statistician and believed that the best way to present this result is with p-values and hope that we can leave it like that.

4. How has the sensitivity, specificity, PPV and NPV established. Describe the gold standard used for comparison.

Reply to reviewer: In this study we calculated the sensitivity, specificity, PPV and NPV of the HINE score for both good outcome (survival and normal/mildly abnormal development at 18 months, respectively) and poor outcome (dead or moderate/severely abnormal development at 18 months), according to standard statistical methods.

5. To describe other studies that have been carried out in LMICs and have looked at short-term outcomes of TH for comparison (Mascarenhas D, Goyal M, Nanavati R, Kirthana SB, Subhadarsini S. Short-term outcome and complications of therapeutic hypothermia in neonates with moderate-to-severe hypoxic ischaemic encephalopathy: a single-centre retrospective observational study in a hospital in Mumbai, India. Paediatr Int Child Health. 2023 Feb 8:1-10. doi: 10.1080/20469047.2023.2171762. Epub ahead of print. PMID: 36755411.)

Reply to reviewer: We thank the reviewer for suggesting the study by Mascarenhas et al. and have added it as a reference in the Discussion. The study has many similarities with our population (similar cooling method [PCM] and aEEG results) although there are many important differences as well (mainly inborn population, many children small for gestational age, a majority (87%) had moderate HIE, and lower mortality). To make comparisons between the studies easer we have added results of short-term outcomes including shock, PPHN, sepsis during hospitalization from our study and discussed this and the comparison in the discussion section.

6. Hearing and vision assessments results not described in the results section

Reply to reviewer: Thank you for this comment, we have now added the hearing and vision assessment results.

7. When was cerebral palsy diagnosed? Not clear in methodology

Reply to reviewer: CP was diagnosed at 18 months. We have added this information in the method section.

8. The neurological impairment described are substantially high hence needs more description of therapeutic hypothermia protocol in the unit

Reply to reviewer: The hypothermia protocol in the unit is described in the Methods section. We have added that the initial management and Apgar scores were not known in many infants. Many infants also had severe encephalopathy, as assessed on arrival and verified by very depressed initial aEEGs, which we believe has contributed to the adverse outcomes.

9. Conclusion needs to be modified in abstract as current study has not evaluated risk factors for NDI.

Reply to reviewer: We have rewritten the Conclusion in both the abstract and the main article. The conclusion in the abstract now reads: “The rates of mortality and adverse neurodevelopment rate were high and comparable to recently published data from other low-middle income settings. The ASQ and HINE were useful tools for evaluation of neurodevelopment and neurological function.” And in the main article this now reads: “The rates of mortality and adverse neurodevelopment in this low-middle income setting were high and comparable to results from other similar settings. Although not formally evaluated, we found that the ASQ and HINE were useful tools for early evaluation of neurodevelopment and neurological function in these children.”
10. Data on CFM recordings
Reply to reviewer: CFM recordings are an important part of following up children with TH. We have added the initial CFM/aEEG results as baseline information in Table 1.

Reviewer: 2
I wonder why more sophisticated analyses weren't done on such useful data. Having scores at multiple time points would let you trace the path of each baby and you could use multilevel models to look at whether any covariates affect that path, or the sensitivity, specificity etc. If cooling worked better for boys, or something like that, that would be very good to know.

But I won't insist on it.

Reply to reviewer: Thank you for this suggestion. However, we have discussed this issue with the study statistician and believed that the cohort is too small to make comparisons between the subgroups.

The analysis in Table 1 needs to be described in the text and mentioned in a footnote to the table.
Reply to reviewer: We have revised Table 1, and added the statistical analyses, and also included data on the infants who died in the neonatal period.

In Table 2 at 6 months, how can mild delay be better than normal? Is this a typo? If not, it needs discussion. It also does not match figure 3.
Reply to reviewer: We apologize. It was a typo, and has now been corrected. Thank you for noting this.

It would also be nice to have figures showing infants over time. You could show time on the X axis and score on the Y axis and a line for each child. With your N you might want to divide this into two or more figures, either randomly or by some sensible criteria.
Reply to reviewer: Thank you for this idea. We have added Figure 4: The individual infants' HINE scores from 6 to 18 months.

Reviewer: 3

Title: Please mention what outcomes – short or long term, Neurodevelopmental to be specific in the title.

Reply to reviewer: The study looked at medium-term outcome (18 months postnatal age). We have made changes accordingly, so the title now reads "Therapeutic hypothermia after perinatal asphyxia in Vietnam - medium-term outcomes at 18 months: a prospective cohort study". As you can see we have also added "a prospective cohort study" based on input from the editor (please see above).

Abstract:
Line 51-52: Conclusion: Mortality and risk for adverse neurodevelopment were relatively high but comparable to other cohorts. Comparable to what cohorts ??

Reply to reviewer: We have discussed and compared the mortality and adverse neurodevelopment rate with other studies in the discussion section. We have now changed the sentence to clarify what comparisons that have been done so the Conclusions now reads: "The rates of mortality and adverse neurodevelopment rate were high and comparable to recently published data from other low-middle income settings. The ASQ and HINE were useful tools for evaluation of neurodevelopment and neurological function."

Line 53-56 The ASQ and HINE are helpful tools in the process of early diagnosis of infants at risk for cerebral palsy or delayed neurodevelopment.
Kindly stick to the results. The study is not about ASQ/HINE validation so remove this line and better to stick to the outcomes please. If you want to mention them in your objectives which doesn't state the same.

Reply to reviewer: Based on comments from the reviewers, we have updated the aims of the study, so that part now reads: "To determine neurodevelopmental outcome at 18 months after therapeutic hypothermia for hypoxic-ischemic encephalopathy (HIE) infants in Vietnam, a low-middle income country."

Introduction : Needs updating, use HIE or

Reply to reviewer: We use the term hypoxic-ischemic encephalopathy (HIE) throughout the manuscript and introduce the abbreviation in the abstract, and also added it the first time it is mentioned in the main text. (in Aims in abstract and first sentence in Introduction "Prematurity, infections, and hypoxic-ischemic encephalopathy (HIE), which results from a lack of oxygen and blood

flow to the brain after delivery, are the three leading causes of mortality among newborns".

Methods : Need more details
- Type of PCM used (Company and manufacturer name)

Reply to reviewer: In this study we used a PCM mattress from Medical Cooling Sweden AB; by TST AB, Kinna, Sweden. We have added this information in the Methods section, so it now reads "…. a mattress made of phase-change material (PCM) (Medical Cooling Sweden AB; by TST AB, Kinna, Sweden)".

- Inclusion criteria as per TOBY used blood gas. Does the study centre used Blood gas for all infants ??

Reply to reviewer: In the TOBY trial the A criteria stated that the infant should have a low <=5) Apgar score at 10 minutes and require resuscitation at 10 minutes, and an umbilical cord pH<7.0 and BD =>16 mmol/L. The B criteria specified signs of HIE, and C criteria for depressed electrocortical activity or seizures (aEEG). In this study all infants were referred from smaller delivery units. Apgar scores were not always given, but on telephone consultation before transfer, the attending neonatologist in Hanoi checked that the patient history was compatible with these requirements, except for the umbilical cord blood gas, which could not be analyzed in the delivery units. B and C criteria were assessed on arrival in Hanoi.

Results
- Baseline characteristics of all infants who underwent cooling needs to be presented total of 130 infants. The baseline data of survivors can be different table . or babies who died versus who survived which gives some info on the phenotype of HIE presentation and severity.

Reply to reviewer: We have followed your advice and made changes in Table 1, now including baseline characteristic of all 130 recruited infants, dead or survived.

Table 1 : Please highlight the significant P values example : Neonatal seizures p<0.05 etc as legend
Reply to reviewer: We have highlighted the significant P values with a *

Is the P value given between two groups or three groups – Was ANOVA used ? if so mention in the legend

Reply to reviewer: Yes, the potential differences between the four groups were analyzed with a one-way ANOVA test. We have added this in the legend.

Use categorization of MRI abnormalities which are standard for HIE staging by Seeta Shankaran et al Shankaran S, McDonald SA, Laptook AR, et al. Neonatal magnetic resonance imaging pattern of brain injury as a bio- marker of childhood outcomes following a trial of hypothermia for neonatal hypoxic-ischemic encephalopathy. J Pediatr. 2015;167(5):987-993.e3. OR Diggikar S, Venkatagiri P, Kulkarni S, Sharma D, Chandra P. Clinical and Neuroimaging Profile of Neonates With Moderate-to-Severe Encephalopathy Treated With Therapeutic Hypothermia. Journal of Neonatology. 2022;36(4):268-274. doi:10.1177/09732179221130671

Reply to reviewer: Unfortunately, it is not possible to re-score the MRIs at this stage according to Shankaran et al. Our MRI knowledge has improved during recent years with the assistance of

international experts from abroad. The MRIs were systematically interpreted for clinical use and our classification was simplified. The assessed MRI features agree very well with the reporting in the systematic review and meta-analysis by Mathew et al. (J Global Health 2022), which has been added to the reference list.

Discussion : Overall discussion is vague, need to be more specific. Needs modification in language and grammar.

Reply to reviewer: We have revised the discussion and then had it reviewed by an English professional editor again. We hope that the reviewers (and all readers) find it better organized.

Conclusion : Doesn't match the objectives stated. Needs to be changes

Reply to reviewer: The conclusion has been updated and it now reads: "The rates of mortality and adverse neurodevelopment in this low-middle income setting were high and comparable to results from other similar settings. Although not formally evaluated, we found that the ASQ and HINE were useful tools for early evaluation of neurodevelopment and neurological function in these children.

VERSION 2 – REVIEW

**REVIEWER**	Dr. Shivshankar Diggikar<br>Ovum Women and Child Hospital, Neonatology
**REVIEW RETURNED**	01-Nov-2023

**GENERAL COMMENTS**	Well done for the changes. Draft looks much better now.

VERSION 2 – AUTHOR RESPONSE

None
